# METTL3 inhibits inflammation of retinal pigment epithelium cells by regulating NR2F1 in an m^6^A-dependent manner

**DOI:** 10.3389/fimmu.2022.905211

**Published:** 2022-07-21

**Authors:** Jiayu Meng, Xianyang Liu, Shiyun Tang, Yusen Liu, Chenyang Zhao, Qian Zhou, Na Li, Shengping Hou

**Affiliations:** ^1^ The First Affiliated Hospital of Chongqing Medical University, Chongqing, China; ^2^ Chongqing Key Laboratory of Ophthalmology, Chongqing Eye Institute, Chongqing, China; ^3^ Ophthalmology, Chongqing Branch of National Clinical Research Center for Ocular Diseases, Chongqing, China; ^4^ College of Basic Medicine, Chongqing Medical University, Chongqing, China

**Keywords:** METTL3, RPE, inflammation, M^6^A, NR2F1

## Abstract

N^6^-metyladenosine (m^6^A) RNA methylation has been proven to be involved in diverse biological processes, but its potential roles in the development of lipopolysaccharide (LPS) induced retinal pigment epithelium (RPE) inflammation have not been revealed. In this study, we explored the effects and underlying mechanisms of methyltransferase-like 3 (METTL3) in LPS stimulated RPE cells. Proliferation of METTL3-silenced RPE cells was examined by Cell counting kit-8 (CCK8) and 5-Ethynyl-2´-Deoxyuridine (Edu). Expression of tight junction proteins ZO-1 and Occludin, and secretion of inflammatory factors interleukins (IL)-1, 6 and 8 were detected by Western blotting or Enzyme-linked immunosorbent assay (ELISA). RNA sequencing and methylated RNA immunoprecipitation (MeRIP) sequencing were used to analyze the target gene nuclear receptor subfamily 2 group F member 1 (NR2F1) of METTL3. Our results showed that both human RPE (hRPE) cells and ARPE19 cells exhibited inhibited proliferation, tight junction protein expression, and increased inflammatory factor secretion after METTL3 silencing. Mechanistically, we found that NR2F1, as a METTL3-methylated target gene, inhibits Occludin level and promotes IL-6 secretion of RPE cells in an m^6^A-dependent manner. Interestingly, NR2F1 deficiency reversed the decreased Occludin expression and increased IL-6 secretion in METTL3-defective RPE cells. In conclusion, our study revealed that METTL3 attenuates RPE cell inflammation by methylating NR2F1, suggesting the critical role of METTL3 in RPE cells.

## Introduction

Retinal pigment epithelium (RPE) cells, as the monolayer and polygonal cells, are located between the retina and the choroidal vascular layer and have a variety of functions, such as phagocytosis, barrier, secretion, transport and vitamin A metabolism (1, 2).

A key function of RPE cells is to maintain the integrity of the photoreceptors and visual function. In autoimmune uveitis, the outer blood-retinal barrier (BRB) composed of RPE cells is severely damaged as a direct consequence of lymphocytic infiltration (3–6). Activated leukocytes adhered to retinal vascular plexus followed by upregulation of the adhesion molecules ICAM-1 and P-selectin, and then resulted in increased vascular permeability (7). Simultaneously, the breakdown of BRB caused leukocytes extravasation and recruitment into the retinal parenchyma. In addition, a previous study revealed that RPE cells secrete a series of cytokines, such as IL-6, in lipopolysaccharide (LPS)-induced inflammatory responses in many ocular pathological conditions (8). This study has demonstrated that LPS induced inflammation of RPE cells play a crucial role in initiating ocular inflammation and causing many intraocular inflammatory diseases such as uveitis and age-related macular degeneration (AMD).

N6-methyladenosine (m^6^A), as the most prevalent internal modification on eukaryotic mRNA, is mainly localized in the RRACH motifs (R=G/A, H=A/U/C) (9, 10). In 2011, the identification of fat mass and obesity-associated protein (FTO) as a genuine demethylase of m^6^A modification indicated that m^6^A is a reversible and dynamic RNA modification (11). The m^6^A process is regulated by multiple protein complexes termed “writers”, “erasers” and “readers”. The writer complex, which deposits m^6^A in mRNA, comprises methyltransferase-like 3 and 14 (METTL3 and METTL14) heterodimers and other adaptors (12–14). m^6^A-binding proteins, named readers, are involved in various physiological processes, such as splicing, nuclear export, transcription, translation, degradation and stability (15–17).

Previous studies mostly focused on the role of m^6^A in cancers, while current studies revealed that the dysfunction of m^6^A in macrophages is also a pathogenic factor in inflammatory diseases. Studies have demonstrated that METTL3 attenuates LPS-induced inflammation of macrophages through NF-κB in patients with rheumatoid arthritis (18, 19). Moreover, a regulatory role of m^6^A exists in other inflammatory diseases, such as systemic lupus erythematosus (SLE) and scleroderma (20–22).

However, whether m^6^A is involved in the LPS induced inflammatory response of RPE cells remains unclear, and the purpose of our research is presented here. Our data first revealed that the expression of Occludin was significantly decreased and that the secretion of inflammatory factors, including IL-1β, IL-6 and IL-8, was significantly increased in the METTL3-silenced RPE cells. Furthermore, we found that NR2F1, as a downstream target of METTL3, decreased Occludin expression and directly promoted IL-6 secretion in an m^6^A-dependent manner. In summary, we proved that METTL3 performs a crucial function in LPS stimulated RPE inflammatory response and unveiled the molecular mechanism.

## Results

### Decreased mRNA levels but increased protein expression of METTL3 in RPE cells after LPS stimulation

**Figure 1:**
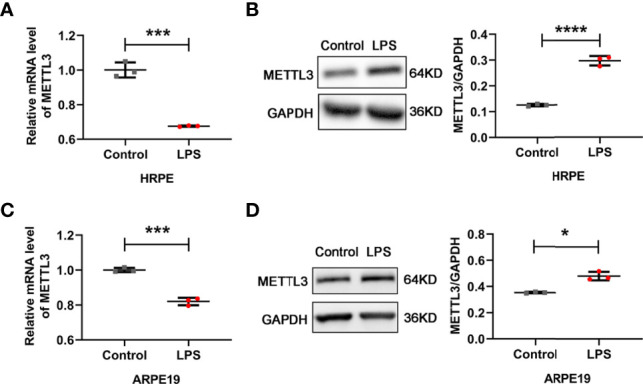
Expression of METTL3 in RPE cells after LPS stimulation. **(A, C)** mRNA level of METTL3 in LPS treated hRPE and ARPE19 cells (mean ± SD; *** P<0.001; n=3/group; unpaired Student’s t-test). **(B, D)** Protein level of METTL3 in LPS treated hRPE and ARPE19 cells. Left, representative western blotting images of METTL3. Right, quantification of relative expression of METTL3 (mean ± SD; * P<0.05, **** P<0.0001; n=3/group; unpaired Student’s t-test).

Given the critical effects of the methyltransferase METTL3 on inflammatory diseases, we detected the expression of METTL3 in human RPE cells and ARPE19 (an RPE cell line) cells (23, 24). Decreased mRNA levels but increased protein expression was found in both hRPE and ARPE19 cells after treatment with LPS, which revealed that METTL3 may play a similar important role in LPS induced RPE autoinflammatory response (Figure 1).

### Silencing METTL3 inhibits proliferation, Occludin expression but promotes IL-6 secretion of hRPE cells

**Figure 2:**
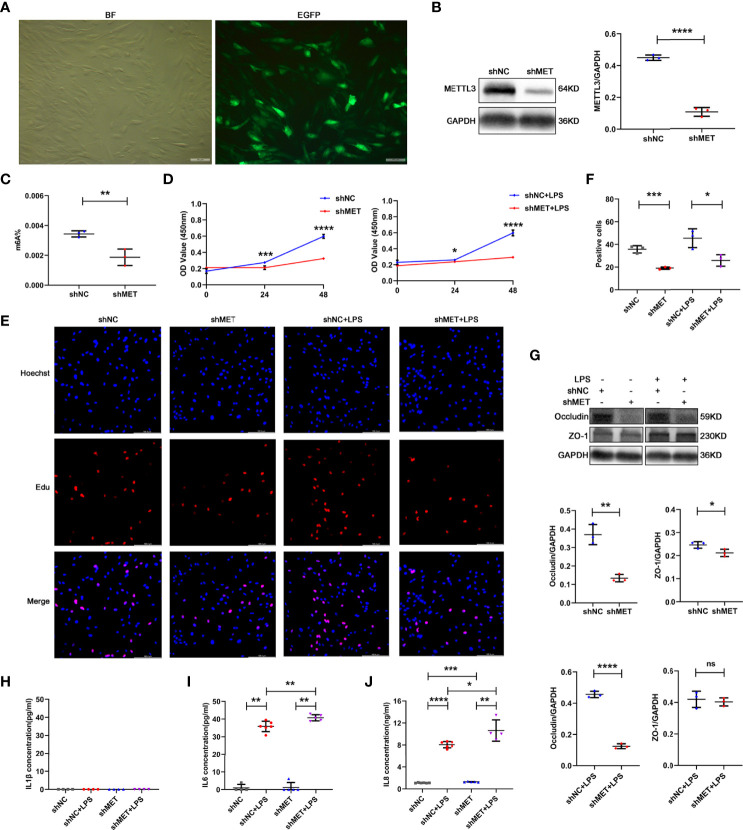
Phenotype of hRPE cells after silencing METTL3. **(A)** Transfection efficiency of the METTL3-silenced lentivirus in hRPE cells (BF, bright field; EGFP, enhanced green fluorescence protein; scale bar, 10 μm). **(B)** Silencing efficiency of METTL3 in hRPE cells. Left, representative western blotting images of METTL3. shMET, shMETTL3; shNC, Negative Control. Right, quantification of relative expression of METTL3 (mean ± SD; **** P<0.0001; n=3/group; unpaired Student’s t-test). **(C)** m^6^A% after silencing METTL3 in hRPE cells (mean ± SD; ** P<0.01; n=3/group; unpaired Student’s t-test). **(D)** Cell viability of hRPE cells after silencing METTL3 with or without LPS stimulation (mean ± SD; * P<0.05, *** P<0.001, **** P<0.0001; n=6/group; unpaired Student’s t-test). **(E, F)** Proliferation of hRPE cells. **(E)** Representative Edu images of hRPE cells after silencing METTL3 with or without LPS stimulation (scale bar, 166.4 μm). **(F)** Quantification of Edu positive cells (mean ± SD; * P<0.05, *** P<0.001; n=3/group; one-way ANOVA). **(G)** Protein levels of Occludin and ZO-1 in hRPE cells after silencing METTL3 with or without LPS stimulation. Upper, representative western blotting images of Occludin and ZO-1. Lower, quantification of relative expression of Occludin and ZO-1 (mean ± SD; ns P>0.05, * P<0.05, ** P<0.01, **** P<0.0001; n=3/group; unpaired Student’s t-test). **(H–J)** The secretion levels of IL-1β, IL-6 and IL-8 in hRPE cells after silencing METTL3 with or without 24 hours of LPS stimulation (mean ± SD; * P<0.05, ** P<0.01, *** P<0.001, **** P<0.0001; n=4-6/group; one-way ANOVA).

To determine the role of METTL3 in RPE cells, we first examined RPE functions by knocking down METTL3 using a METTL3-silencing lentivirus in hRPE cells. After transfection, approximately 90% infection efficiency was observed in these hRPE cells (Figure 2A). Then, we selected stably transformed strains using puromycin and detected the silencing efficiency. Significantly decreased METTL3 expression and m^6^A modification rates were found in the METTL3-silenced cells compared with the control cells (Figures 2B, C). We further explored the changes in hRPE phenotypes, such as proliferation, tight junction protein expression, and secretion, after silencing METTL3. Surprisingly, the proliferation of hRPE cells was decreased in the METTL3-silenced cells with or without LPS stimulation (Figures 2D–F). The significantly decreased protein level of Occludin was found in the METTL3-silenced hRPE cells (Figure 2G). Finally, we also assessed the secretion function of hRPE cells. Increased secretion of IL-6 was found in the METTL3-silenced hRPE cells with LPS stimulation but not found in the cells without LPS stimulation. The secretion of IL-8 in METTL3-silenced hRPE cells with LPS stimulation was increased more than that without LPS stimulation, which confirmed the increased inflammation of the METTL3-silenced hRPE cells (Figures 2H–J).

### Silencing METTL3 inhibits proliferation, tight junction protein expression but promotes IL-6 secretion in ARPE19 cells

**Figure 3:**
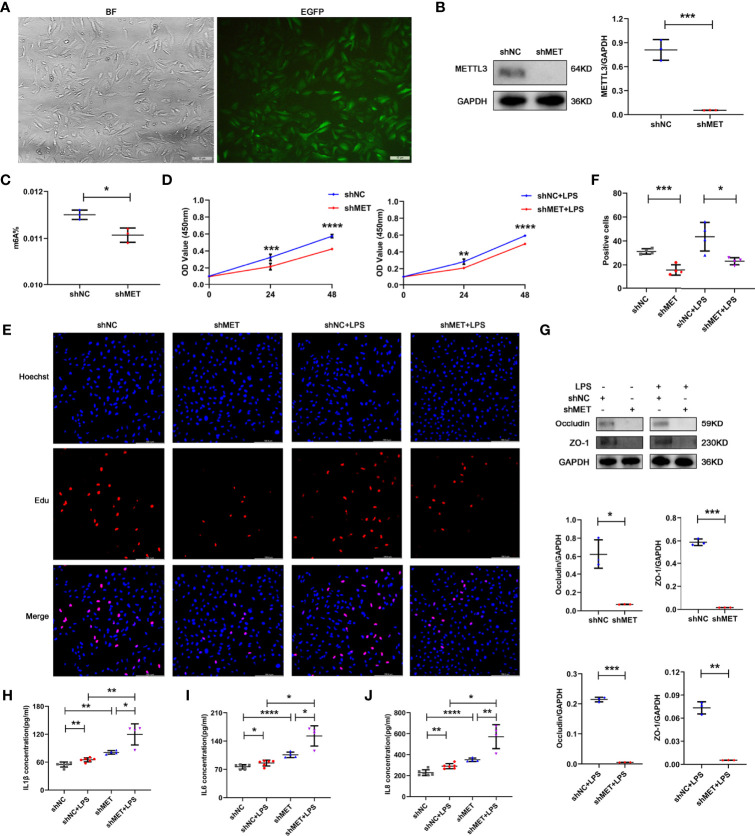
Phenotype of ARPE19 cells after silencing METTL3. **(A)** Transfection efficiency of the METTL3-silenced lentivirus in ARPE19 cells (BF, bright field; EGFP, enhanced green fluorescence protein; scale bar, 10 μm). **(B)** Silencing efficiency of METTL3 in ARPE19 cells. Left, representative western blotting images of METTL3. Right, quantification of relative expression of METTL3 (mean ± SD; *** P<0.001; n=3/group; unpaired Student’s t-test). **(C)** m^6^A% after silencing METTL3 in ARPE19 cells (mean ± SD; * P<0.05; n=3/group; unpaired Student’s t-test). **(D)** Cell viability of ARPE19 cells after silencing METTL3 with or without LPS stimulation (mean ± SD; ** P<0.01, *** P<0.001, **** P<0.0001; n=5-6/group; unpaired Student’s t-test). **(E, F)** Proliferation of ARPE19 cells. **(E)** Representative Edu images of ARPE19 cells after silencing METTL3 with or without LPS stimulation (scale bar, 166.4 μm). **(F)** Quantification of Edu positive cells (mean ± SD; * P<0.05, *** P<0.001; n=4/group; one-way ANOVA). **(G)** Protein levels of Occludin and ZO-1 in ARPE19 cells after silencing METTL3 with or without LPS stimulation. Upper, representative western blotting images of Occludin and ZO-1. Lower, quantification of relative expression of Occludin and ZO-1 (mean ± SD; * P<0.05, ** P<0.01, *** P<0.001; n=3/group; unpaired Student’s t-test). **(H–J)** The secretion levels of IL-1β, IL-6 and IL-8 in ARPE19 cells after silencing METTL3 with or without 24 hours of LPS stimulation (mean ± SD; * P<0.05, ** P<0.01, **** P<0.0001; n=4-6/group; one-way ANOVA).

To further confirm the role of METTL3 in RPE cells, we also examined RPE functions by silencing METTL3 in ARPE19 cells. Similarly, approximately 90% infection efficiency was observed in the ARPE19 cells after transfection with lentivirus (**Figure 3A**). Then, we also selected the stably transformed strains and detected the silencing efficiency. Decreased expression of METTL3 and decreased m^6^A modification rates were also found in the METTL3-silenced ARPE19 cells (Figures 3B, C). In addition, the proliferation of ARPE19 cells was decreased in the METTL3-silenced cells with or without LPS stimulation (Figures 3D–F). However, the protein levels of both Occludin and ZO-1 were significantly decreased in the METTL3-silenced ARPE19 cells, which was different from the METTL3-silenced hRPE cells (Figure 3G). Finally, the secretion of IL-1β, IL-6 and IL-8 was shown to be increased in the METTL3-silenced ARPE19 cells especially with LPS stimulation (Figures 3H–J).

### METTL3 epigenetically represses NR2F1 in an m^6^A-dependent manner

**Figure 4:**
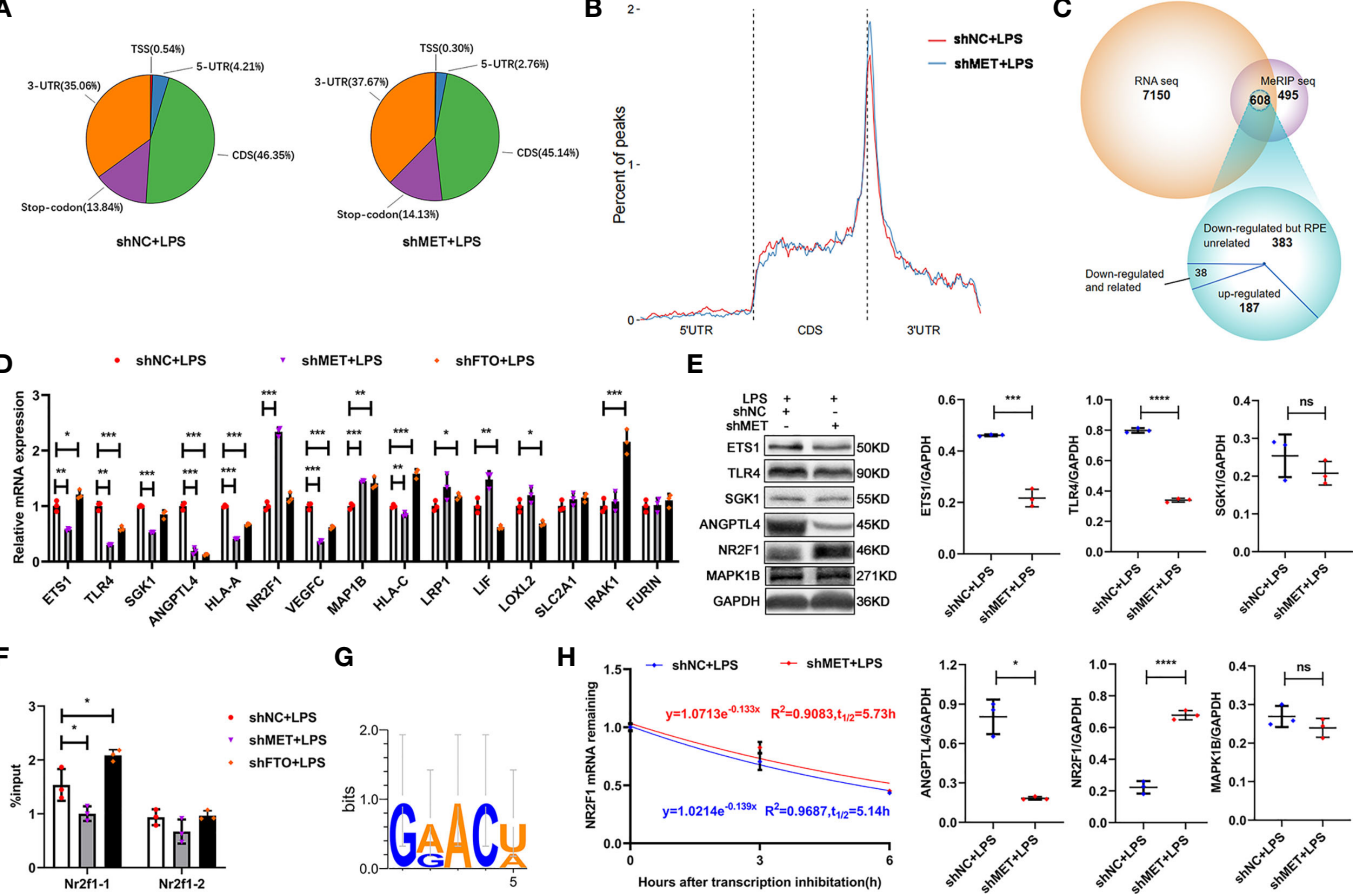
METTL3 inhibits NR2F1 in an m6A-dependent manner. **(A)** Pie charts represent the percent of all m^6^A peaks on mRNA (CDS, coding sequence; UTR, untranslated region; TSS, transcription start site). **(B)** The percent of m^6^A peaks on three segments of mRNA: 5´UTR, CDS and 3´UTR. **(C)** Venn diagram of MeRIP-seq and RNA-seq to identify common differentially expressed genes (DEG). **(D)** mRNA levels of ETS1, TLR4, SGK1, ANGPTL4, HLA-A, NR2F1, VEGFC, MAP1B, HLA-C, LRP1, LIF, LOXL2, SLC2A1, IRAK1 and FURIN in the METTL3- or FTO-silenced ARPE19 cells after LPS stimulation (mean ± SD; * P<0.05, ** P<0.01, *** P<0.001; n=3/group; one-way ANOVA). **(E)** Protein levels of ETS1, TLR4, SGK1, ANGPTL4, NR2F1 and MAP1B in the METTL3-silenced ARPE19 cells after LPS stimulation. Left, representative western blotting images of these proteins. Right, quantification of relative expression (mean ± SD; ns P>0.05, * P<0.05, *** P<0.001, **** P<0.0001; n=3/group; unpaired Student’s t-test). **(F)** Site-specific mRNA levels of NR2F1 after m^6^A immunoprecipitation in the METTL3- or FTO-silenced ARPE19 cells (mean ± SD; * P<0.05; n=3/group; one-way ANOVA). **(G)** NR2F1 binding motif identify. **(H)** The half-life detection of NR2F1 in the METTL3-silenced ARPE19 cells after LPS stimulation (mean ± SD; n=3/group).

To investigate how METTL3 affects the inflammation of RPE cells, we performed RNA sequencing and methylated RNA immunoprecipitation (MeRIP) sequencing using the METTL3-silenced ARPE19 cells stimulated with LPS. The results showed that most of the m^6^A modifications were located in the coding sequence (CDS) and 3´untranslated region (UTR) of ARPE19 cell mRNA in ARPE19 cells regardless of METTL3 silencing (Figures 4A, B). To identify the critical target genes, we divided the 608 common genes from these two sequencings into three parts: 38 m^6^A modifications-downregulated and RPE cell function-related genes, 383 m^6^A modifications-downregulated but RPE cell function-unrelated genes, and 187 m^6^A modifications-upregulated genes (Figure 4C). From these 38 genes with downregulated m^6^A modifications and RPE cell function-related, 15 RPE inflammation-related genes, such as ETS1, were selected for verification. Significantly increased expression of NR2F1 was found in the METTL3-silenced ARPE19 cells, which was positively related to its proinflammatory function (Figures 4D, E) (25). Then, we verified the m^6^A modification and analyzed the motif of NR2F1 mRNA. However, only one modification site could be verified in the METTL3- and FTO-silenced ARPE19 cells after LPS stimulation, and further motif analysis found that this m^6^A site may be GG(/A)ACU(/A) (Figures 4F, G). Finally, we detected the mRNA half-life of NR2F1, and a longer half-life was observed in the METTL3-silenced ARPE19 cells than in the control cells (Figure 4H).

### Silencing NR2F1 inhibits inflammation of RPE cells

**Figure 5:**
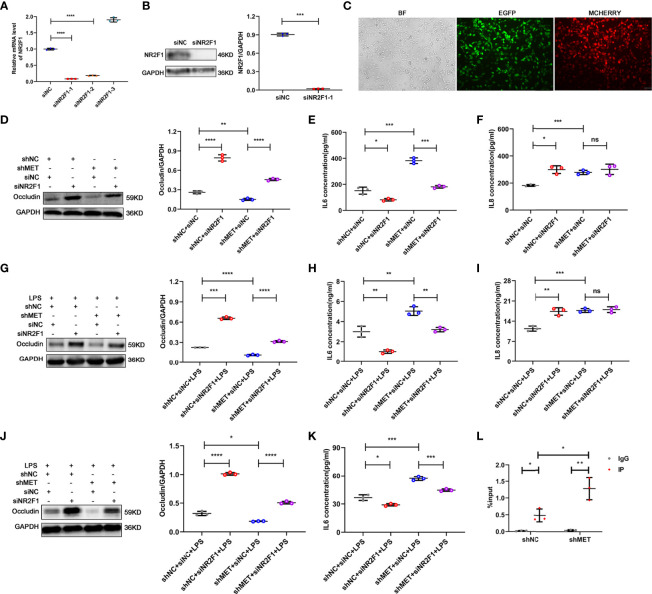
Silencing NR2F1 increased expression of Occludin and inhibited secretion of IL-6. **(A)** Fold changes of NR2F1 mRNA after silencing (mean ± SD; **** P<0.0001; n=3/group; one-way ANOVA). **(B)** Silencing efficiency of NR2F1 using siNR2F1-1. Left, representative western blotting images of NR2F1. Right, quantification of relative expression of NR2F1 (mean ± SD; *** P<0.001; n=3/group; unpaired Student’s t-test). **(C)** Cotransfection of METTL3- and NR2F1-silenced lentivirus (BF, bright field; EGFP, enhanced green fluorescence protein; MCHERRY, red fluorescence protein; scale bar, 10μm). **(D)** Protein level of Occludin after METTL3 and NR2F1 silencing in ARPE19 cells. Left, representative western blotting images of Occludin. Right, quantification of relative expression of Occludin (mean ± SD; ** P<0.01, **** P<0.0001; n=3/group; one-way ANOVA). **(E, F)** The secretion levels of IL-6 and IL-8 in ARPE19 cells after METTL3 and NR2F1 silencing (mean ± SD; ns P>0.05, * P<0.05, *** P<0.001; n=3/group; one-way ANOVA). **(G)** Protein level of Occludin in the METTL3- and NR2F1-silenced ARPE19 cells with LPS stimulation. Left, representative western blotting images of Occludin. Right, quantification of relative expression of Occludin (mean ± SD; *** P<0.001, **** P<0.0001; n=3/group; one-way ANOVA). **(H, I)** The secretion levels of IL-6 and IL-8 in ARPE19 cells with LPS stimulation after METTL3 and NR2F1 silencing (mean ± SD; ns P>0.05, ** P<0.01, *** P<0.001; n=3/group; one-way ANOVA). **(J)** Protein level of Occludin after METTL3 and NR2F1 silencing in hRPE cells with LPS stimulation. Left, representative western blotting images of Occludin. Right, quantification of relative expression of Occludin (mean ± SD; * P<0.05, **** P<0.0001; n=3/group; one-way ANOVA). **(K)** The secretion level of IL-6 in hRPE cells with LPS stimulation after METTL3 and NR2F1 silencing (mean ± SD; * P<0.05, *** P<0.001; n=3/group; one-way ANOVA). **(L)** The DNA expression of IL-6 after METTL3 silencing with NR2F1 immunoprecipitation (IgG for negative control; mean ± SD; * P<0.05, ** P<0.01; n=3/group; one-way ANOVA).

To verify the effect of NR2F1 on RPE cell inflammation, we silenced NR2F1 in the METTL3-silenced RPE cells. First, we examined the transfection efficiency after lentiviral infection and silencing efficiency after selecting the stably transformed strains with puromycin and neomycin. Approximately 90% infectious efficiency and silencing efficiency were observed in the METTL3 and NR2F1 double-silenced RPE cells (Figures 5A–C). Next, we detected the expression of Occludin and secretion of IL-6 and IL-8 to evaluate the effect of NR2F1 on RPE inflammation. The results showed that the expression of Occludin was increased in the NR2F1-silenced ARPE19 cells and rescued in the METTL3 and NR2F1 double-silenced ARPE19 cells with or without LPS stimulation (Figures 5D, G). The secretion of IL-6 was decreased in the NR2F1-silenced ARPE19 cells and rescued in the METTL3 and NR2F1 double-silenced ARPE19 cells (Figures 5E, H). Surprisingly, the secretion of IL-8 was increased in the NR2F1-silenced ARPE19 cells but not rescued in the METTL3 and NR2F1 double-silenced ARPE19 cells, which suggested that IL-8 could be regulated by METTL3 and NR2F1 but not the METTL3/NR2F1 pathway (Figures 5F, I). Similar changes in Occludin and IL-6 were observed in the hRPE cells after LPS stimulation (Figures 5J, K). To further investigate how NR2F1 regulates Occludin and IL-6, we performed binding prediction for transcription factors and promoters (Bioinfo.life.hust.edu.cn/hTFtarget#)! and then verified that NR2F1 could directly bind to the promoter of IL-6 (Figure 5L).

## Discussion

RNA methylation, as an emerging posttranscriptional modification of RNA, has attracted increasing attention among epigenetic modifications, especially m^6^A. Previous studies have demonstrated that m^6^A, the most abundant mRNA internal modification, regulates diverse cellular processes in cancer, immunology, and other human diseases (26–28). However, whether m^6^A is involved in LPS stimulated inflammatory response of RPE cells is still not clear. At the beginning of our research, we found that the protein expression of the methylase METTL3 was increased in both hRPE and ARPE19 cells after LPS stimulation, which revealed the potential role of METTL3 in RPE cell inflammation and laid the foundation for subsequent studies.

Our studies showed decreased proliferation, tight junction protein expression, but increased inflammatory factor secretion in METTL3-silenced RPE cells. Mechanistically, we proposed for the first time that NR2F1, a transcription factor methylated by METTL3, regulates Occludin expression and IL-6 secretion of RPE cells. In addition, silencing of NR2F1 rescued the inflammatory phenotype in the METTL3-silenced RPE cells, suggesting that METTL3 regulates the RPE inflammation *via* the NR2F1/IL-6 pathway. However, no studies have shown the transcriptional regulation of IL-6 by NR2F1.

The nuclear cotranscriptional regulator NR2F1, also named COUP-TF1, is an orphan nuclear receptor belonging to the superfamily of the steroid/thyroid hormone receptors (29). Previous studies have shown that NR2F1 is mainly implicated in brain cell cycle control, cancer cell dormancy, invasion and metastasis (30). Its proteostasis was modulated by insufficiency of RAN-binding protein-2 (RANBP2) in a post-translational modification upon oxidative stress in RPE cells (29). However, the specific modification is still unknown. In our results, we suggested that NR2F1 was epigenetically regulated by METTL3 in an m^6^A-dependent manner, which paved the way for further studies on the epigenetic modification of NR2F1.

Given the increased protein expression of METTL3 and obvious phenotypic differences have been shown in our results after silencing METTL3 in RPE cells, we tried to verify these conclusions in some models. Surprisingly, we found that the level of m^6^A%, methylase METTL3 and demethylase FTO were all decreased in primary RPE cells of experimental autoimmune uveitis (EAU) mice (Supplementary Figure 1 and unpublished data). In addition, these decreased METTL3 expression in RPE cells of EAU or endotoxin induced uveitis (EIU) mice is contrary to the *in vitro* findings, which revealed a complex process with various cells interaction of the *in vivo* models.

**Figure 6:**
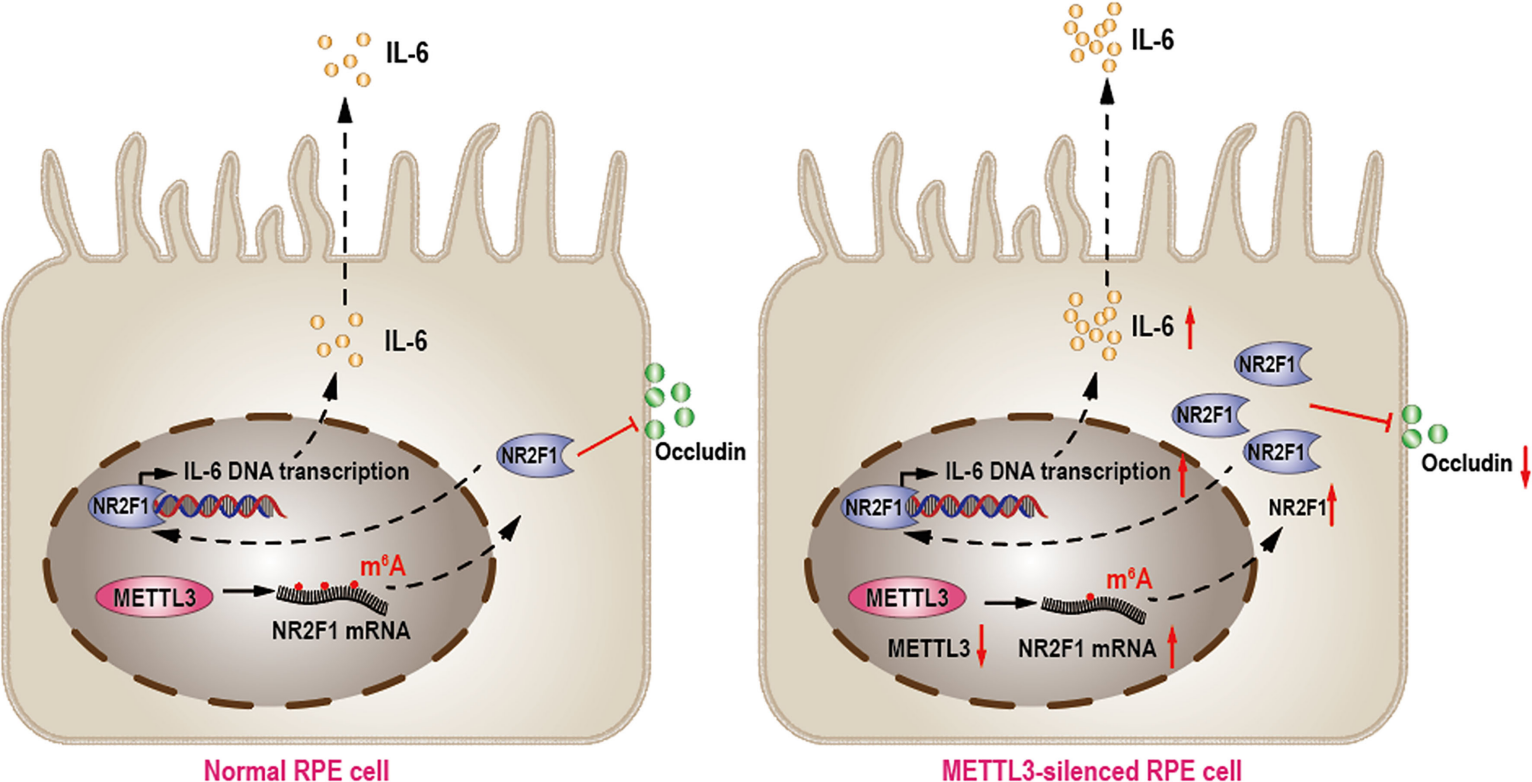
METTL3 regulates RPE inflammation by NR2F1 in an m^6^A-dependent manner. In normal RPE cell, METTL3 methylates NR2F1 and then decreases protein level of NR2F1, which lead to reduced IL-6 transcription and secretion but increased Occludin expression (Left). In METTL3-silenced RPE cell, NR2F1 is increased and then promotes IL-6 secretion but inhibits Occludin expression, which finally lead to increased inflammation (Right).

In summary, our results showed that METTL3 regulated RPE cell inflammation *in vitro* by methylating NR2F1 in an m^6^A-dependent manner (Figure 6).

## Materials and methods

### Cell culture

The ARPE19 cell line was purchased from the American Type Culture Collection (CRL-2302, ATCC, USA) and hRPE cells were obtained from donors visited at the First Affiliated Hospital of Chongqing Medical University. All the eyeball samples were collected from the patients with written informed consent and the procedures were approved by the Ethics Committee of the First Affiliated Hospital of Chongqing Medical University (2019-099). The posterior ocular tissues within 24 hours after death were rinsed 3 times with sterile phosphate buffer solution (PBS) and then digested by 0.25% trypsin containing EDTA (25200072, Gibco, USA) at 37°C for 30 min. After filtered a 40 μm cell strainer, the single hRPE cells were washed 3 times again with PBS and then cultured in Dulbecco’s Modified Eagle Medium: F-12 (DMEM/F-12, 11320033, Gibco, USA) with 10% fetal bovine serum (FBS, 10100147, Gibco, USA) and 1% Penicillin-Streptomycin solution (15070063, Gibco, USA) in the humidified incubator at 37°C with 5% CO2.

### Cell stimulation and lentiviral infection

Cells were stimulated with 1 μg/ml LPS (L2880, Sigma-Aldrich, USA) for 24 h to induce inflammation and infected with lentivirus carrying shMETTL3 or siNR2F1 packaged by Genechem Co., Ltd. (Shanghai, China) or Sangon Biological Engineering Technology & Services Ltd. Co. (Shanghai, China) according to the manufactures’ instructions. Briefly, ARPE19 or hRPE cells were sowed and cultured overnight, then infected with lentivirus (multiplicity of infection, MOI: 30) for 6 h. On day 3 after transfection, cells were observed and images were collected using a fluorescence microscope (DMIL4000, Leica, Germany). And then, the stably transformed strains were selected by puromycin or neomycin for this study.

### Real-time quantitative PCR

Total RNA was extracted from RPE cells using the Trizol reagent (Roche, Swiss) according to the manufacturer’s instructions. RNA concentration was measured by spectrophotometer (Thermo Fisher Scientific. Inc., MA, USA), and cDNA was synthesized with the RT Master Mix for qPCR reagent (MedChemExpress, USA). Real-time quantitative polymerase chain reaction (RT-qPCR) was performed using the ABI 7500 Real-time PCR System (Applied Biosystems, CA, USA) with SYBR Green qPCR Master Mix (MedChemExpress, USA) according to the manufacturer’s instructions. All primers sequences can be found in Supplementary Table 1. Relative expression was normalized to β-actin and calculated using the 2^−ΔΔCT^ method.

### Western blotting

Cells were lysed in radio immunoprecipitation assay (RIPA, Beyotime, Shanghai, China) lysis buffer containing 1% Phenylmethanesulfonyl fluoride (PMSF, Beyotime, Shanghai, China) on ice and the proteins were quantified with the bicinchoninic acid assay quantification kit (BCA, Beyotime, Shanghai, China). Samples were separated by sodium dodecyl sulfate polyacrylamide gel electrophoresis (SDS-PAGE), and then electrotransferred onto polyvinylidene difluoride membranes (PVDF, Millipore, Billerica, MA, USA). After being blocked in 5% non-fat milk for 2 h at room temperature, the membranes were subjected to immunoblotting with primary antibodies at 4°C overnight. Following incubated with secondary antibodies at room temperature for 1 h, the blots were visualized using the ECL kit (Mengbio, Chongqing, China). The density of bands was quantified by Image J, and normalized to GAPDH. Primary antibodies used in this study are provided in Supplementary Table 2.

### m^6^A RNA quantification assay

Total RNA was extracted using the Trizol reagent (Roche, Swiss) and the m^6^A RNA content was measured using the EpiQuik m^6^A RNA Methylation Quantification kit (Colorimetric, Epigentek, Farmingdale, NY, USA) according to the manufacturer’s instructions. In short, 200 ng of sample RNA was coated on an assay well for the analysis after added with binding solution. Followed with detection antibody and capture antibody, enhanced solution was added to incubate. Finally, optical density (OD) values of wells at 450 nm were measured and calculated based on negative and positive controls.

### Cell counting kit-8 (CCK8) assay

RPE cells were seeded into 96-well plates (5 × 10^3^ cells/well) and cultured for 0, 24, and 48 h at 37°C. For proliferation detection, the original culture medium was replaced with medium containing CCK-8 reagent (Invigentech, CA, USA) for another 2 h. The absorbance was measured using a microplate reader at 450 nm (Thermo Fisher Scientific. Inc. MA, USA).

### 5-ethynyl-2´-deoxyuridine (Edu) staining

Proliferation of RPE cells was investigated with BeyoClick™ Edu-555 Cell Proliferation Detection Kit (C0075S, Beyotime, China) according to the manufacturer’s protocols. Briefly, after being seeded into 12-well plates for 24 h at 37°C, RPE cells were incubated with Edu working solution (10 µM) for 2 h. Then washed with PBS twice and fixed with 4% paraformaldehyde for 15 min at room temperature. Next, the cells were permeabilized with 0.3% TritonX-100 (Beyotime, China) for 10 min and washed with PBS three times. Finally, cells were incubated with Click Additive Solution for 30 min in dark, and subsequently stained with 1X Hoechst for the nucleus staining. Images were captured with the fluorescence microscope (Leica, Germany). RPE cells at DNA replication phase appeared red fluorescence while the nuclei represented blue fluorescence.

### Enzyme-linked immunosorbent assay (ELISA)

The supernatant of RPE cells with or without 24 hours of LPS stimulation was collected, and then the concentration of inflammatory cytokines (IL-1β, IL-6 and IL-8) was measured using Elisa kits (Elabscience, Wuhan, China) according to the manufacturer’s instructions. The microplate reader (Thermo Fisher Scientific. Inc. MA, USA) was used to detect the absorbance values at the wavelength of 450 nm.

### Methylated RNA immunoprecipitation-sequencing (MeRIP-seq) and PCR

The procedure of m^6^A immunoprecipitation (MeRIP) was performed on the basis of previously reported methods (31–33). In brief, purified mRNAs were fragmented into approximately 100 nt of length by a 45 s incubation at 94°C in RNA fragmentation reagent (AM8740, Life Technologies). Then, the fragmented mRNAs were collected and incubated with anti-m^6^A antibody or IgG in immunoprecipitation buffer containing RNase inhibitor at 4°C overnight. Methylated RNAs were immunoprecipitated with protein A/G beads (Life Technologies, 10013D), eluted by competition with free m^6^A and for library construction or qPCR analysis.

### RNA-sequencing

Total RNA was extracted from RPE cells using the Trizol reagent as mentioned above. For mRNA-sequencing, mRNAs were single-end sequenced on Illumina HiSeq 2000 machines in Novogene Technology Co., Ltd. (Beijing, China). Transcript assembly and differential expression was examined by Cufflink with Refseq mRNAs.

### mRNA stability assay

After LPS stimulation, control and METTL3 knockdown RPE cells were treated with 5 μg/mL actinomycin D (A1410, Sigma-Aldrich, USA) for 0, 3, and 6 h. Cells were harvested at each time point and subjected to RNA extraction. RT-qPCR was used to examine the mRNA abundance of target genes in each group.

### Chromatin immunoprecipitation (ChIP)-qPCR

The ChIP assay was performed using the Chromatin Immunoprecipitation Kit (17-295, Millipore, Germany) according to the manufacturer’s instructions. Briefly, RPE cells were cross-linked with 1% formaldehyde (12606S, Cell Signaling technology) for 10 min, and quenched with glycine for 5 min at room temperature. Then, cells were collected, washed, and resuspended in lysis buffer. Then cross-linked DNAs were fragmented with sonication (6% energy, 30 s for 6 cycles). The sonicated chromatin solution was incubated with beads coated with 5 μg of anti-NR2F1 (ab181137, abcam, UK) or IgG at 4°C overnight. Immunoprecipitated DNAs were purified and analyzed by qPCR.

### Statistical analyses

Comparison between two groups was analyzed by two-tailed Student’s t-test, and comparison among three or more groups was analyzed by one-way ANOVA. The differences analyzed by SPSS software 20.0 (IBM, USA) were considered to be statistically significant at P<0.05. Data are presented as mean ± SD, and figures were made using Prism version 8.0 software (GraphPad, San Diego, USA).

## Data availability statement

The datasets presented in this study can be found in online repositories. The name of the repository and accession number can be found below: NCBI Gene Expression Omnibus; GSE202018.

## Ethics statement

The studies involving human participants were reviewed and approved by the Ethics Committee of the First Affiliated Hospital of Chongqing Medical University (2019-099). The patients/participants provided their written informed consent to participate in this study.

## Author contributions

JM and XL designed the research, performed the experiments and wrote the manuscript; ST, YL, CZ, and QZ helped to perform the experiments; NL and SH helped to design the research and revise the manuscript. All authors have read and approved the final manuscript.

## Funding

This study was supported by the National Natural Science Foundation Project of China (82070951 and 81873678); the Innovative Research Group Project of Chongqing Education Commission (CXQT19015); the Natural Science Foundation Project of Chongqing (cstc2019jcyjmsxmX0120); the Innovation Supporting Plan of Overseas Study of Chongqing (cx2018010); the Chongqing Education Commission (KJQN202000406); the National Key Clinical Specialties Construction Program of China, Chongqing Branch of National Clinical Research Center for Ocular Diseases; the Chongqing Key Laboratory of Ophthalmology (CSTC, 2008CA5003) and Program for Youth Innovation in Future Medicine, Chongqing Medical University (w0047).

## Conflict of interest

The authors declare that the research was conducted in the absence of any commercial or financial relationships that could be construed as a potential conflict of interest.

## Publisher’s note

All claims expressed in this article are solely those of the authors and do not necessarily represent those of their affiliated organizations, or those of the publisher, the editors and the reviewers. Any product that may be evaluated in this article, or claim that may be made by its manufacturer, is not guaranteed or endorsed by the publisher.
