# Supplementary material for: METTL3 inhibits inflammation of retinal pigment epithelium cells by regulating NR2F1 in an m6A-dependent manner
Source: Front Immunol. 2022 Jul 21;13:905211. doi: 10.3389/fimmu.2022.905211 (PMC9351451; doi:10.3389/fimmu.2022.905211)
Supplement: Supplementary file 1 [file DataSheet_1.docx]

**Supplementary information**

**Supplementary figure 1 Decreased expression of METTL3 in RPE cells of EAU or EIU mice.**

1. m^6^A% of EAU mice.

**B.** mRNA levels of m^6^A writers, erasers and readers in EAU mice.

**C.** The protein expression of METTL3 in EAU mice.

**D.** The protein expression of METTL3 in EIU mice.


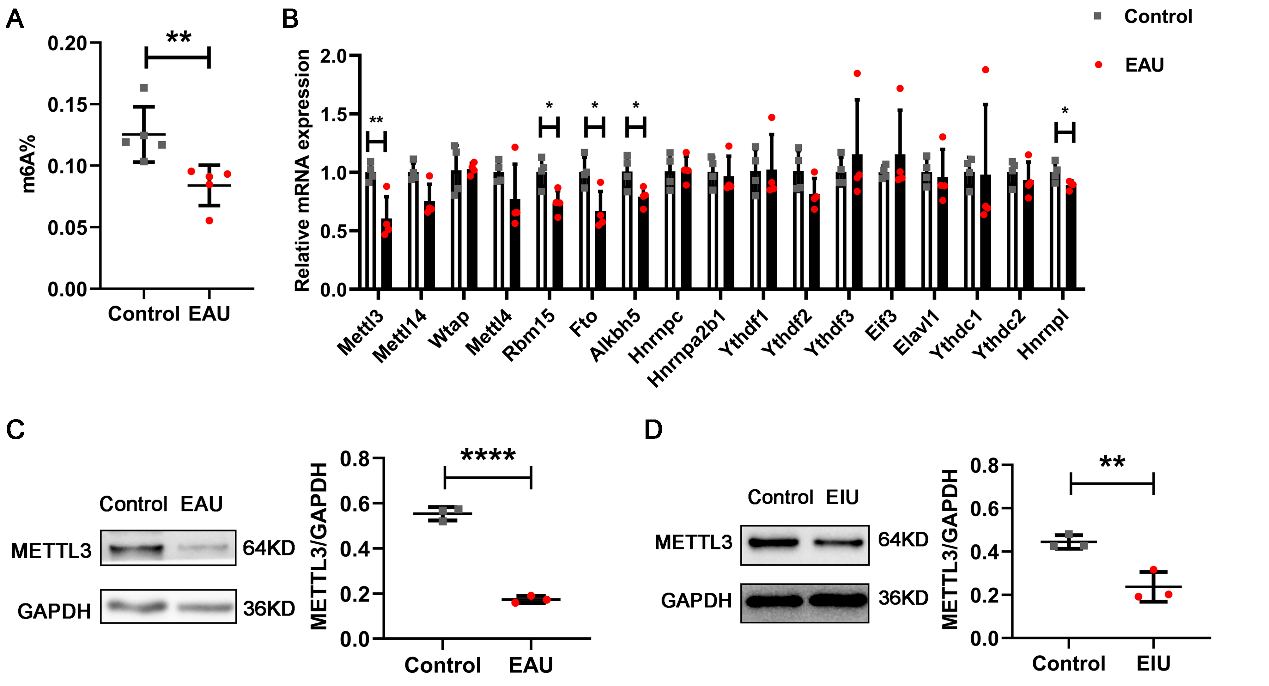


**Supplementary table 1 Primers for RT-qPCR.**

| **Genes** | **Species** | **Sequences (5’-3’)** |
| --- | --- | --- |
| METTL3 | Human | TTGTCTCCAACCTTCCGTAGT |
|  |  | CCAGATCAGAGAGGTGGTGTAG |
| β-ACTIN | Human | GGATGCAGAAGGAGATCACTG |
|  |  | CGATCCACACGGAGTACTTG |
| ETS1 | Human | GATAGTTGTGATCGCCTCACC |
|  |  | GTCCTCTGAGTCGAAGCTGTC |
| TLR4 | Human | AGACCTGTCCCTGAACCCTAT |
|  |  | CGATGGACTTCTAAACCAGCCA |
| SGK1 | Human | CATATTATGTCGGAGCGGAATGT |
|  |  | TGTCAGCAGTCTGGAAAGAGA |
| ANGPTL4 | Human | GGCTCAGTGGACTTCAACCG |
|  |  | CCGTGATGCTATGCACCTTCT |
| HLA-A | Human | AAAAGGAGGGAGTTACACTCAGG |
|  |  | GCTGTGAGGGACACATCAGAG |
| NR2F1 | Human | ATCGTGCTGTTCACGTCAGAC |
|  |  | TGGCTCCTCACGTACTCCTC |
| VEGFC | Human | GAGGAGCAGTTACGGTCTGTG |
|  |  | TCCTTTCCTTAGCTGACACTTGT |
| MAP1B | Human | ATCTCGACACTCTGCAAGATTCT |
|  |  | TGTTTCTAAAACGTCACTTCGGT |
| HLA-C | Human | CCATGAGGTATTTGTGGACCG |
|  |  | TCTCGGACTCTCGTCGTCG |
| LRP1 | Human | CTGACTGGCGAACAAACACA |
|  |  | ACGGTCCGGTTGTAGTTGAT |
| LIF | Human | TGAACCAGATCAGGAGCCAA |
|  |  | GCCAAGGTACACGACTATGC |
| LOXL2 | Human | GGGTGGAGGTGTACTATGATGG |
|  |  | CTTGCCGTAGGAGGAGCTG |
| SLC2A1 | Human | CATGGGCTTCTCGAAACTGG |
|  |  | GGTCCTTGTTGCCCATGATG |
| IRAK1 | Human | GCACCCACAACTTCTCGGAG |
|  |  | CACCGTGTTCCTCATCACCG |
| FURIN | Human | GCAAAGCGACGGACTAAACG |
|  |  | TGCCATCGTCCAGAATGGAGA |
| NR2F1-1 | Human | AAGCACTACGGCCAATTCAC |
|  |  | ACTTCTTGAGGCGGCAGTAT |
| NR2F1-2 | Human | ACCTTGTGTCTGTCTGGTGA |
|  |  | GGCTTCAGTCCACTTCCATATG |

**Supplementary table 2 Antibodies for western blotting.**

| **Antibody** | **Host** | **Manufacturer** | **Application** |
| --- | --- | --- | --- |
| METTL3 | Rabbit | abcam | 1:1,000 |
| GAPDH | Mouse | Proteintech | 1:10,000 |
| ZO-1 | Rabbit | Proteintech | 1:500 |
| Occludin | Mouse | Invitrogen | 1:1,000 |
| ETS1 | Rabbit | abcam | 1:1,000 |
| TLR4 | Rabbit | abcam | 1:1,000 |
| SGK1 | Rabbit | abcam | 1:500 |
| ANGPTL4 | Rabbit | abcam | 1:1,000 |
| NR2F1 | Rabbit | abcam | 1:1,000 |
| MAP1B | Rabbit | abcam | 1:1,000 |
